# *COMT1* Silencing Aggravates Heat Stress-Induced Reduction in Photosynthesis by Decreasing Chlorophyll Content, Photosystem II Activity, and Electron Transport Efficiency in Tomato

**DOI:** 10.3389/fpls.2018.00998

**Published:** 2018-07-17

**Authors:** Golam J. Ahammed, Wen Xu, Airong Liu, Shuangchen Chen

**Affiliations:** ^1^College of Forestry, Henan University of Science and Technology, Luoyang, China; ^2^Department of Horticulture, Guizhou University, Guiyang, China

**Keywords:** chlorophyll fluorescence, heat stress, JIP test, melatonin, photosynthesis, tomato

## Abstract

Despite a range of initiatives to reduce global carbon emission, the mean global temperature is increasing due to climate change. Since rising temperatures pose a serious threat of food insecurity, it is important to further explore important biological molecules that can confer thermotolerance to plants. Recently, melatonin has emerged as a universal abiotic stress regulator that can enhance plant tolerance to high temperature. Nonetheless, such regulatory roles of melatonin were unraveled mainly by assessing the effect of exogenous melatonin on plant tolerance to abiotic stress. Here, we generated melatonin deficient tomato plants by silencing of a melatonin biosynthetic gene, *CAFFEIC ACID O-METHYLTRANSFERASE 1* (*COMT1*), to unveil the role of endogenous melatonin in photosynthesis under heat stress. We examined photosynthetic pigment content, leaf gas exchange, and a range of chlorophyll fluorescence parameters. The results showed that silencing of *COMT1* aggravated heat stress by inhibiting both the light reactions and the carbon fixation reactions of photosynthesis. The photosynthetic pigment content, light absorption flux, trapped energy flux, energy dissipation, density of active reaction center per photosystem II (PSII) cross-section, the photosynthetic electron transport rate, the maximum photochemical efficiency of PSII photochemistry, and the rate of CO_2_ assimilation all decreased in *COMT1*-silenced plants compared with that of non-silenced plants particularly under heat stress. However, exogenous melatonin alleviated heat-induced photosynthetic inhibition in both genotypes, indicating that melatonin is essential for maintaining photosynthetic capacity under stressful conditions. These findings provide genetic evidence on the vital role of melatonin in photosynthesis and thus may have useful implication in horticultural crop management in the face of climate change.

## Introduction

Despite a range of initiatives to reduce global carbon emission, the mean global temperature is increasing over time due to climate change. The occurrences of drought and heat events have become more prevalent in recent times, which pose a serious threat to global food security (Field et al., 2014). It is anticipated that the growing season temperatures will exceed the recorded highest seasonal temperatures of the last century by the end of the twenty-first century in the tropics and subtropics. Furthermore, a 1°C increase in seasonal temperature potentially causes 2.5–16% direct yield losses (Battisti and Naylor, 2009). However, a plant can only experience heat shock when the growth temperatures exceed a critical threshold for a period which is sufficient to cause irreversible damage to plants (Ahammed et al., 2016). Notably, photosynthesis is one of the vital physiological processes of plants, highly sensitive to temperature change (Tan et al., 2011). Exposure of plants to high temperature stress decreases the rate of photosynthesis and the amount of photosynthetic pigments in leaves (Shanmugam et al., 2013). Heat stress also causes water loss in aerial plant parts and impairs membrane integrity (Li et al., 2015; Li H. et al., 2016). Both photosystems I and II (PSI and PSII) are affected by heat stress due to the heat-induced inhibition of light energy absorption, energy distribution, and electron transport (Li H. et al., 2016). However, PSII is more sensitive to heat stress than PSI as heat stress can cause severe damage to the reaction center-binding protein D1 of PSII (Yoshioka et al., 2006; Yan et al., 2013). Since heat stress poses a serious threat to global food security, it is important to further explore key biological molecules that can mediate thermotolerance in plants.

Melatonin (*N*-acetyl-5-methoxytryptamine) is a multi-regulatory biological molecule, ubiquitous across the plant and animal kingdom (Arnao and Hernandez-Ruiz, 2018). Before the discovery of melatonin in plants in 1995, this molecule was considered as a unique animal hormone. In recent years, a tremendous progress has been made in unraveling the role of melatonin in plant growth, development, and stress responses (Reiter et al., 2015; Wang et al., 2017; Arnao and Hernandez-Ruiz, 2018). In particular, melatonin is considered as a universal abiotic stress regulator in plants (Wang et al., 2017). It can alleviate a range of abiotic stresses such as heat, cold, drought, salinity, and heavy metals in plants (Zhang et al., 2015; Li M.Q. et al., 2016; Li X. et al., 2016; Xu et al., 2016; Cai et al., 2017; Li et al., 2018b; Qi et al., 2018). However, such conclusions are mostly based on the effect of exogenous melatonin on plants under perturbed environmental conditions. Exogenous melatonin application increases leaf chlorophyll content and suppresses heat-induced leaf senescence in perennial ryegrass (Zhang et al., 2017). Similarly, exogenous melatonin improves photosynthetic carbon assimilation under low temperature in tomato plants by inducing a gene that encodes a Calvin cycle enzyme, sedoheptulose-1,7-bisphosphatase (Ding et al., 2017). Melatonin treatment also reduces heat stress-induced pollen abortion in tomato plants (Qi et al., 2018). In addition, exogenous melatonin improves photosynthetic energy transport efficiency, chlorophyll concentration and the activities of RuBisCO and ATPases in wheat under nano-ZnO-induced stress (Zuo et al., 2017). However, the relevance of endogenous melatonin in plant photosynthetic responses to heat stress has not been substantiated. Therefore, it is indispensable to better understand how manipulation of endogenous melatonin levels influences the photosynthetic responses of plants to heat stress.

Photosynthesis plays an important role in global carbon cycling (Reddy et al., 2010). It involves two core sets of reactions, such as the light reactions and the carbon fixation reactions. Through light reactions, light (electromagnetic) energy is absorbed by photosynthetic pigments followed by conversion into chemical energy (ATP and NADPH), whereas the resulting ATP and NADPH are utilized in the carbon fixation reactions to fix atmospheric carbon dioxide in order to produce sugars. Both light and carbon fixation reactions are sensitive to environmental cues such as light, temperature, and water, and thus changes in those parameters impair the balance between the production of ATP and NADPH, and consumption of these metabolites (Porcar-Castell et al., 2014; Li H. et al., 2016). Since photosynthesis largely determines the plant growth and productivity, reduction of photosynthesis due to extreme temperature eventually reduces crop yield. The rate of CO_2_ fixation can be measured efficiently by monitoring CO_2_ exchange in the enclosed chamber of infrared gas analyzer, whereas chlorophyll fluorescence measurements are used to assess photochemical reactions (Porcar-Castell et al., 2014).

Chlorophyll *a* (Chl *a*) fluorescence intensity of dark-adapted photosynthetic plant parts follows a characteristic variation with time after the onset of illumination (Strasser et al., 2004). The analysis of Chl *a* fluorescence is considered as a sensitive method for the detection and quantification of stress-induced changes in the photosynthetic apparatus (Li et al., 2018a). For decades, chlorophyll fluorescence measurements have been used as non-invasive, rapid and easy to use methods to study the effect of heat stress on photosynthesis in plants (Rapacz, 2007; Brestic and Zivcak, 2013; Porcar-Castell et al., 2014; Li H. et al., 2016). However, the use of maximal photochemical efficiency of PSII photochemistry (*F*_v_/*F*_m_) is the most common in this regards. *F*_v_/*F*_m_ represents amount of energy trapped in PSII reaction centers with regard to energy absorbed (Force et al., 2003). The term photoinhibition is often used to refer a significant decrease in *F*_v_/*F*_m_ and frequently used to analyze heat-induced damage to PSII (Porcar-Castell et al., 2014). Nonetheless, the term photoinhibition also indicates the damage to the reaction center. Meanwhile, the JIP test has been developed for the measurement of polyphasic fluorescence transient which made possible to calculate different types of phenomenological and biophysical expressions of PSII, such as absorption of light energy fluxes, trapping of absorbed energy fluxes, electron transport efficiency, and thermal dissipation (Force et al., 2003). In sweet sorghum, heat stress significantly increases J step in the Chl *a* fluorescence transient, implying that heat stress can inhibit electron transport beyond primary quinone of PSII (Yan et al., 2013). However, it is largely unknown whether and how endogenous melatonin deficiency influences those multiple chlorophyll fluorescence parameters under heat stress in tomato. As an attempt to unveil the role of endogenous melatonin in photosynthesis under heat stress, we generated melatonin deficient tomato plants by silencing a gene of melatonin biosynthesis pathway, *CAFFEIC ACID O-METHYLTRANSFERASE 1* (*COMT1*). We examined photosynthetic pigment content, gas exchange and a range of chlorophyll fluorescence parameters. The results showed that silencing of *COMT1* aggravated heat stress in tomato by inhibiting the light reactions as well as the carbon fixation reactions as reflected by the reduced light energy absorption and distribution, electron transport efficiency, quantum yield of photochemistry, and net CO_2_ assimilation. Our results support the notion that melatonin is essential for maintaining photosynthetic activity under high temperature in tomato plants.

## Materials and Methods

### Plant Materials and Growth Conditions

In the current study, we used tomato (*Solanum lycopersicum* L.) cultivar Ailsa Craig as background plant material for generating melatonin deficient tomato plants. To assess tomato thermotolerance, a group of tomato seedlings at five-leaf stage were kept at 40°C temperature in growth chambers for 9 h. However, the control plants were kept under normal temperature conditions. To examine the effect of exogenous melatonin on photosynthesis, plants were pretreated (8 h prior) with 10 μmol L^−1^ melatonin on leaves before imposition of the heat stress. Ten micromolar working solution was prepared by dissolving the melatonin (Sigma-Aldrich, St. Louis, MO, United States) in ethanol followed by dilution with Milli-Q water [ethanol:water (v:v) = 1:10,000]. The concentration of melatonin was selected based on our previous study on thermotolerance in tomato plants (Xu et al., 2016). The growth conditions, prior to high temperature stress, were as follows: photoperiod of 14/10 h (day/night), temperature of 25/22°C (day/night), and a photosynthetic photon flux density (PPFD) of 800 μmol m^−2^ s^−1^. Plants were fertilized with Hoagland’s nutrient solution every 2 days. Six replicates were performed for each treatment, and each replicate comprised six plants.

### Photosynthetic Pigments and Leaf Gas Exchange Measurements

Photosynthetic pigments such as Chl *a*, chlorophyll *b* (Chl *b*), and carotenoids from third fully expanded leaves were extracted in 80% acetone and the contents (μg g^−1^ FW) were analyzed colorimetrically (Lichtenthaler and Wellburn, 1983). Gas exchange in third fully expanded leaves, including CO_2_ assimilation, stomatal conductance (*G*_s_), intercellular CO_2_ concentration (*C*_i_), and transpiration rate (*T*_r_) were determined using a LI-6400 Portable Photosynthesis System (LI-6400; LI-COR, Lincoln, NE, United States). The light-saturated rate of CO_2_ assimilation (*A*_sat_) was measured by maintaining the air temperature, air relative humidity, CO_2_ concentration, and PPFD at 25°C, 80–90%, 400 μmol mol^−1^, and 1000 μmol m^−2^ s^−1^, respectively.

### Determination of Chlorophyll Fluorescence and JIP Test Parameters

Chlorophyll fluorescence transients were measured after a 15-min dark adaptation and recorded up to 1 s on a logarithmic timescale with a Dual-PAM-100 system (Heinz Walz, Effeltrich, Germany). Data were obtained every 20 μs. The polyphasic fluorescence induction kinetics was analyzed according to the JIP test (Strasser and Govindjee, 1992). Initial fluorescence (*F*_0_) was measured at 20 μs using the fast-rise kinetic curves when all PSII reaction centers (RCs) are open. *F*_300__μs_ is the fluorescence at 300 μs; *F*_J_ and *F*_I_ are the fluorescence intensity at step J (2 ms) and at step I (30 ms), respectively. The maximal fluorescence (*F*_M_) is the peak of fluorescence at the step P when all RCs are closed. Area is total complementary area between fluorescence induction curves. Parameters quantifying PSII behavior such ABS/CSm, TR/CSm, ET/CSm, and D/CSm were calculated from the above original data as mentioned in the Supplementary Table S1 (Strasser and Strasser, 1995; Strasser et al., 2004).

### Measurements of Energy Conversion and Electron Transport in PSI and PSII

A simultaneous measurement of quantum yield of PSI [Y(I)] and PSII [Y(II)] in tomato leaves was performed with a Dual-PAM-100 system (Heinz Walz, Effeltrich, Germany) on the measure mode of Fluo + P700 (Pfündel et al., 2008). *F*_0_, the minimum fluorescence, was monitored under a weak light pulse (<0.1 μmol m^−2^ s^−1^). A saturating pulse (10,000 μmol photons m^−2^ s^−1^) was then applied to obtain the maximum fluorescence after dark adaptation (*F*_m_). The maximum photochemical efficiency of PSII (*F*_v_/*F*_m_) was calculated using the experimentally determined *F*_0_ and *F*_m_, where *F*_v_ was the difference between *F*_0_ and *F*_m_. The maximal change of P700 signal (*P*_m_) was measured through application of a saturation pulse (10,000 μmol photons m^−2^ s^−1^) after illumination of far-red light for 10 s. To determine the maximum fluorescence signal (*F*_m_′) and maximum P700 + signal (*P*_m_′), a saturating pulse with duration of 300 ms was applied every 20 s after the onset of the actinic light (27 μmol photons m^−2^ s^−1^). The slow induction curve was recorded for 300 s to achieve the steady state of the photosynthetic apparatus, and then the actinic light was turned off. After the final saturating pulse, values of Y(II), ETR(II), Y(I), and ETR(I) were recorded for analysis of PSI and PSII activity.

### Virus-Induced Gene Silencing Constructs and *Agrobacterium*-Mediated Virus Infection

The tobacco rattle virus (TRV)-based virus-induced gene silencing (VIGS) construct was used to silence the tomato *COMT1* gene. The 300-bp fragment of *COMT1* gene was PCR amplified from tomato cDNA using the forward and the reverse primers (Supplementary Table S2) containing *Xba*I and *Bam*HI restriction sites. The amplified fragment was digested and ligated into the same TRV2 sites. The correct plasmid was transformed into the *Agrobacterium tumefaciens* GV3101. Then a mixed culture of *A. tumefaciens* carrying the TRV1:TRV2-target gene in a 1:1 ratio was infiltrated into the fully expanded cotyledonary leaves of the seedlings (Li M.Q. et al., 2016). The plants that were infiltrated with *A. tumefaciens* carrying the empty TRV1 and TRV2 vectors were used as controls TRV. The efficiency of VIGS and inhibition of melatonin biosynthesis were confirmed by the analysis of relative expression of *COMT1* and melatonin content in leaves by qRT-PCR and HPLC analysis, respectively as described previously (Li M.Q. et al., 2016; Xu et al., 2016). The inoculated plants exhibited approximately 30% of the transcript levels of the TRV plants confirmed the success of VIGS (Supplementary Figure S1 and Supplementary Table S3).

### Statistical Analysis

At least six replicates were used for each experiment, and the mean values of all the data are presented for each treatment. A statistical analysis of the obtained data was performed with the SPSS 18 statistical software package. The Tukey’s test (*P* < 0.05) was performed to evaluate the treatment effect.

## Results

### Suppression of Melatonin Biosynthesis Inhibits Photosynthetic Pigment Accumulation and Increases Sensitivity to Heat Stress

**FIGURE 1:**
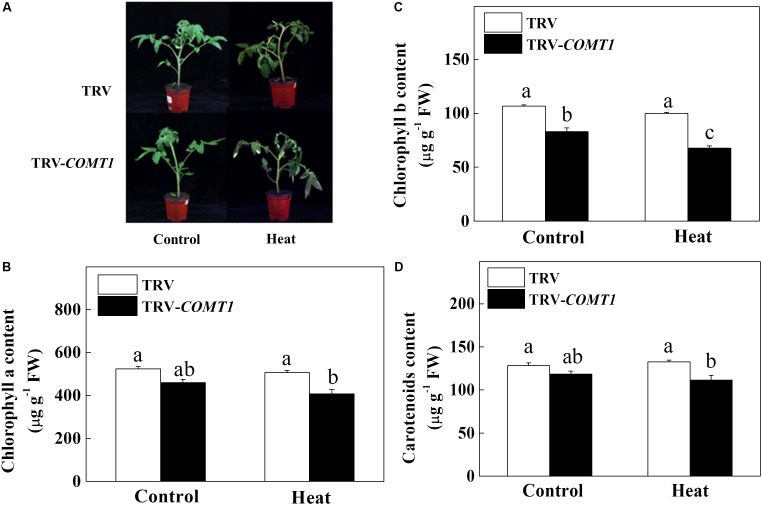
Effects of *COMT1* silencing and heat stress on plant phenotype and photosynthetic pigment concentration. **(A)** Plant phenotype, **(B)** chlorophyll *a* content, **(C)** chlorophyll *b* content, and **(D)** carotenoids content in third fully expanded leaves. Tomato seedlings at five-leaf stage were exposed to 40°C temperature for 9 h before analysis of the phenotype and pigment contents. The results are expressed as the mean values ± SE, *n* = 6. The mean values denoted by the same letter do not significantly differ at a *P* < 0.05 according to the Tukey’s test.

Since exogenous melatonin showed a protective role against heat stress (Qi et al., 2018), we intended to unveil the role of endogenous melatonin in plant tolerance to heat stress. Therefore, we suppressed melatonin biosynthesis by silencing of *COMT1*, which drastically decreased melatonin content in tomato leaves (Supplementary Figure S1). Then we challenged these plants with a 9 h heat stress (40°C) and examined the whole plant phenotype and photosynthetic pigment contents. We noticed obvious sign of heat injuries in the plants that were kept at 40°C compared with those at 25°C (**Figure 1**). However, melatonin deficient TRV-*COMT1* plants were more affected than the TRV control as reflected by the wilting phenotypes. Quantification of photosynthetic pigment contents revealed that silencing of *COMT1* significantly reduced Chl *b* concentration (**Figure 1**). Although the contents of Chl *a*, Chl *b*, and carotenoids were not altered by heat stress in TRV control plants, heat stress significantly decreased the Chl *a*, Chl *b*, and carotenoids contents in TRV-*COMT1* plants compared with that in heat-stressed TRV plants.

### Endogenous Melatonin Deficiency Alters Gas Exchange Under Heat Stress in Tomato Leaves

**FIGURE 2:**
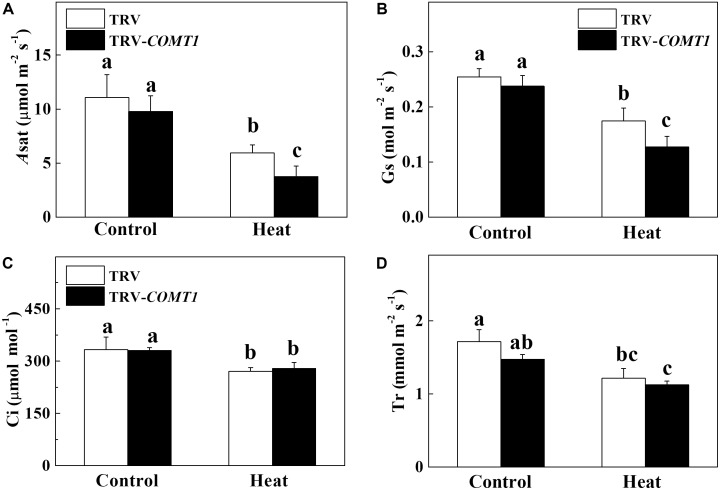
Changes in gas exchange parameters as influenced by *COMT1* silencing and heat stress in tomato leaves. **(A)** The light-saturated rate of CO_2_ assimilation (*A*_sat_), **(B)** stomatal conductance (*G*_s_), **(C)** intercellular CO_2_ concentration (*C*_i_), and **(D)** transpiration rate (*T*_r_). Tomato seedlings at five-leaf stage were exposed to 40°C temperature for 9 h before analysis of gas exchange parameters. Data are presented as the mean of six replicates (±SE). Different letters indicate significant differences (*P* < 0.05) according to the Tukey’s test.

To assess whether *COMT1* silencing affects leaf gas exchange, we measured the *A*_sat_, *G*_s_, *C*_i_, and *T*_r_ in tomato leaves. Under normal temperature conditions, no difference was found between TRV and TRV-*COMT1* plants in terms of *A*_sat_, *G*_s_, *C*_i_, and *T*_r_ (**Figure 2**). However, heat stress drastically decreased *A*_sat_, *G*_s_, *C*_i_, and *T*_r_ in both genotypes. The effect of heat stress was more pronounced in TRV-*COMT1* plants in terms of *A*_sat_ and *G*_s_ values as compared with that of the TRV plants. However, no significant difference was found in *C*_i_ and *T*_r_ values between TRV and TRV-*COMT1* plants under heat stress.

### Endogenous Melatonin Deficiency Affects Energy Absorption and Distribution Fluxes Under Heat Stress

**FIGURE 3:**
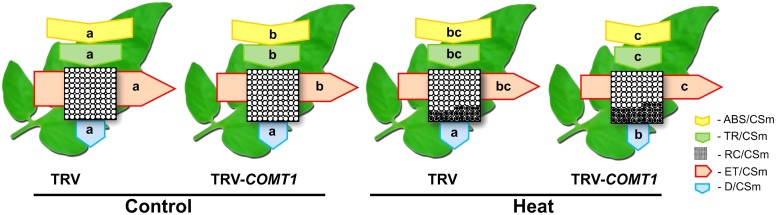
Energy pipeline leaf models of phenomenological fluxes (per cross-section, CS) in the third fully expanded leaf in tomato as influenced by *COMT1* silencing and heat stress. The results are expressed as the mean values ± SE, *n* = 6. Each relative value is drawn by the width of the corresponding arrow, standing for a parameter. Empty and full black circles indicate the percentage of active (Q_A_ reducing) and non-active (non-Q_A_ reducing) reaction centers of photosystem II (PSII), respectively; ABS/CSm, photon flux absorbed by the antenna pigments per CS; TR/CSm, trapped energy flux per CS; ET/CSm, electron transport flux per CS; D/CSm, non-photochemical quenching. Different letters indicate significant differences (*P* < 0.05) according to the Tukey’s test.

To explore how melatonin deficiency affects light energy absorption and distribution, we measured the absorption flux per PSII cross-section (ABS/CSm), the trapped energy flux per PSII cross-section (TR/CSm), the electron transport in PSII cross-section (ET/CSm), the energy dissipation per PSII cross-section (D/CSm), and the density of active reaction centers (RC/CSm) by chlorophyll fluorescence analysis. The obtained data were used to construct the phenomenological pipeline models of energy fluxes which showed that heat stress significantly decreased ABS/CSm, TR/CSm, and E/CSm (**Figure 3**). Although silencing of *COMT1* caused a significant decrease in ABS/CSm, TR/CSm, and E/CSm, heat stress further decreased those parameters. D/CSm remained unchanged in TRV plants, but heat stress significantly decreased D/CSm in TRV-*COMT1* plants. In addition, heat stress decreased the density of active reaction centers as indicated by the reduced number of open circles both in TRV and TRV-*COMT1* plants, whereas the number of closed circles, which indicates the inactive reaction center density was higher in TRV-*COMT1* plants than that in TRV plants under heat stress.

### Endogenous Melatonin Deficiency Influences Photochemical Reactions in PSI and PSII Under Heat Stress

**FIGURE 4:**
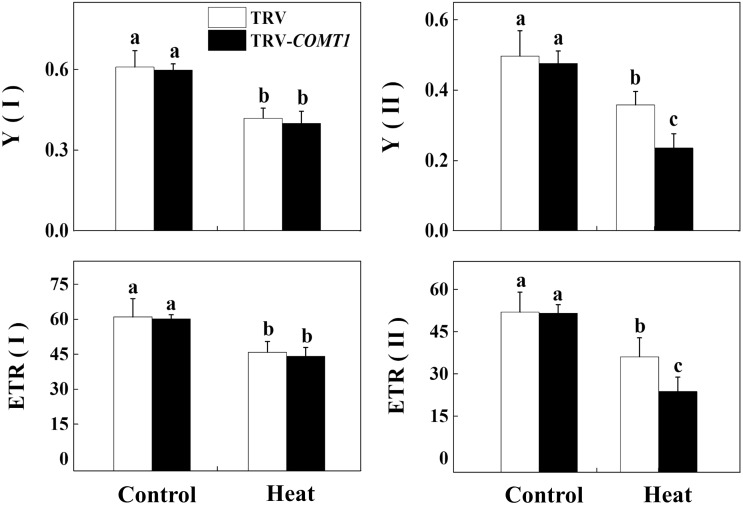
Effect of *COMT1* silencing and heat stress on quantum yield and electron transport rate in PSI and PSII in tomato leaves. Tomato seedlings at five-leaf stage were exposed to 40°C temperature for 9 h before analysis of chlorophyll fluorescence parameters. The bars (means ± SE, *n* = 6) labeled with different letters are significantly different at *P* < 0.05 according to Tukey’s test. Y(I), effective quantum yield of PSI; Y(II), effective quantum yield of PSII; ETR(II), electron transport rate of PSII; ETR(I), electron transport rate of PSI.

Next, we determined the changes in photochemistry of PSI and PSII by using Dual-PAM-100 measuring system that simultaneously assessed energy conversion in both PSI and PSII. Consistent with the changes in *A*_sat_, heat stress decreased Y(II) and Y(I) compared with that of control. Silencing of *COMT1* did not affect Y(II) under normal condition, but these melatonin-deficient plants showed decreased Y(II) under heat stress compared with their TRV counterpart (**Figure 4**). Meanwhile, no significant difference was found in Y(I) value between TRV and TRV-*COMT1* either at normal temperature or heat stress condition. Although the ETR(II) was significantly inhibited by the heat stress in both TRV and TRV-*COMT1* plants, the effect of heat stress was more pronounced on TRV-*COMT1* plants compared with that on the TRV plants. Similar to Y(II), heat stress decreased ETR(II) in both genotypes, but no significant difference of ETR(I) was found between TRV and TRV-*COMT1* under the same temperature conditions.

### Exogenous Melatonin Compensates Endogenous Melatonin Deficiency and Improves Photosynthesis Under Heat Stress

**FIGURE 5:**
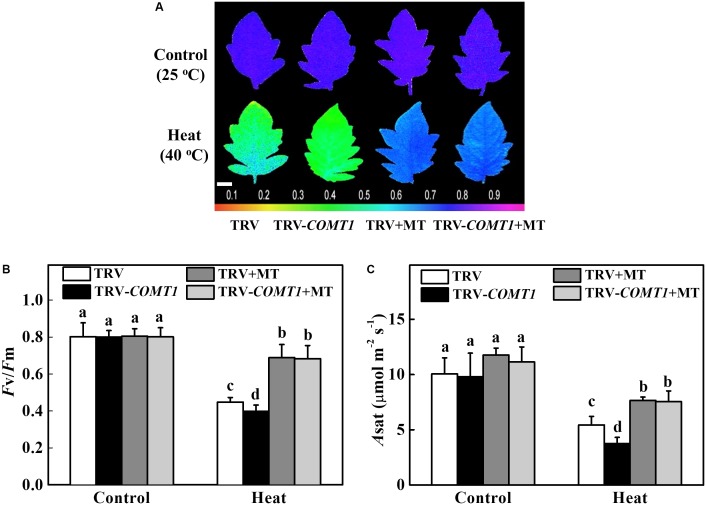
Effects of *COMT1* silencing, exogenous melatonin and heat stress either alone or combined on the maximum photochemical efficiency of photosystem II (*F*_v_/*F*_m_) and CO_2_ assimilation rate. **(A)** *F*_v_/*F*_m_ shown in pseudo color images, the false color code depicted at the bottom of the image ranges from 0 (black) to 1 (purple); **(B)** *F*_v_/*F*_m_ values; and **(C)** the light-saturated rate of CO_2_ assimilation (*A*_sat_). Tomato seedlings at five-leaf stage were exposed to 40°C temperature for 9 h before analysis. Plants were pretreated with 10 μmol L^−1^ melatonin on leaves at 8 h prior to imposition of the heat stress. *F*_v_/*F*_m_ and *A*_sat_ both were measured on the third fully expanded leaves; however, plants were dark adapted for 30 min before recording the *F*_v_/*F*_m_ by using an imaging pulse amplitude modulated (PAM) fluorimeter (IMAG-MAXI; Heinz Walz, Effeltrich, Germany). The results are expressed as the mean values ± SE, *n* = 6. Different letters indicate significant differences (*P* < 0.05) according to the Tukey’s test.

To further confirm the functional relevance of melatonin on photosynthesis, we first treated the foliar portion of the TRV as well as the melatonin deficient TRV-*COMT1* plants with 10 μmol L^−1^ melatonin solution. Eight hours after melatonin treatment, tomato seedlings were exposed to a high temperature regime (40°C) for 9 h. Then we measured gas exchange and chlorophyll fluorescence to evaluate the changes in *A*_sat_ and *F*_v_/*F*_m_, respectively. As shown in **Figure 5**, heat stress significantly decreased *F*_v_/*F*_m_ by 44.18% in TRV plants, which further decreased in TRV-*COMT1* plants under heat stress. On the other hand, exogenous melatonin increased the *F*_v_/*F*_m_ by 53.79 and 71.58% in TRV and TRV-*COMT1* plants compared with the *F*_v_/*F*_m_ in the respective only heat treatment. Similarly, heat stress-induced reductions in CO_2_ assimilation (*A*_sat_) was remarkably attenuated by exogenous melatonin treatment both in TRV and TRV-*COMT1* plants, implying that exogenous melatonin compensated the *COMT1*-silencing-induced melatonin deficiency in tomato plants and resulted in an improved photosynthetic capacity under heat stress.

## Discussion

In general, the non-destructive and timesaving fluorescence measurements of electron transport rate relate well to the quantum efficiency of photosynthesis (Genty et al., 1989). However, a number of factors can influence the shape of such relationship. The relationship between CO_2_ assimilation and fluorescence-based analysis of electron transport rate is largely affected by chlorophyll content and leaf structure (Tsuyama et al., 2003). In the current study, we found slightly decreased chlorophyll contents in *COMT1*-silenced plants, and this phenomenon was profound under heat stress condition, at which *COMT1*-silenced plants accumulated significantly lower levels of photosynthetic pigments than that of TRV plants. In wheat, Chl *b*-deficient mutant lines show decreased CO_2_ assimilation compared to the wild-type under heat stress, suggesting a close association between chlorophyll content and CO_2_ assimilation capacity under heat stress (Brestic et al., 2016). In fact, chlorophyll contents in TRV plants were not affected by this short duration heat stress, which was in agreement with Li H. et al. (2016), who found no difference in leaf chlorophyll contents following a 48 h heat treatment (40°C) in cucumber. Since loss of chlorophyll under perturbed condition accelerates senescence, it is logical to assume that leaves of melatonin deficient *COMT1*-plants will senescence earlier than the TRV plants. This assumption is also based on the fact that exogenous melatonin can delay chlorophyll degradation and improve photosynthetic capacity in plants under abiotic stress (Zhang et al., 2017; Li et al., 2018a). Furthermore, exogenous melatonin improves carbon assimilation in Chl *b*-deficient mutant wheat by increasing total chlorophyll concentration in leaves which indicates the involvement of melatonin in maintaining chlorophyll concentration (Li et al., 2018a). In our study, *COMT1*-silenced plants showed a significantly lower level of Chl *b* than that of TRV plants (**Figure 1**), indicating that melatonin deficiency profoundly affects Chl *b* concentration in tomato leaves.

Photosynthesis is the unique physiological process in plants through which light energy in the photosystem is transformed into chemical energy, and is used for CO_2_ assimilation in the Calvin–Benson cycle (Reddy et al., 2010; Porcar-Castell et al., 2014). Consistent with the changes in chlorophyll content, we found a significantly reduced CO_2_ assimilation capacity in *COMT1*-silenced plants compared with that of TRV plants under heat stress. The decrease in *A*_sat_ was attributed to heat-induced reductions in *G*_s_ and TR in *COMT1*-silenced plants (**Figure 2**). The reduction in *G*_s_ under heat stress would potentially limit CO_2_ supply leading to an enhanced photodamage to PSII via excessive reduction of Q_A_, impaired repair of photodamaged PSII and inhibition of D1 protein synthesis in intact chloroplasts (Takahashi and Murata, 2008; Li H. et al., 2016).

The process of photosynthesis commences with the absorption of light, especially by the chlorophyll molecules. Thus light absorption in a leaf largely depends on the leaf chlorophyll content (Brodersen and Vogelmann, 2010). ABS/CS indicates the number of photons absorbed by an excited PSII cross-section (Force et al., 2003). Since melatonin-deficient plants accumulate slightly decreased chlorophyll content (**Figure 1**), it would be expected that leaves of TRV-*COMT1* plants should have relatively lower absorptance. Indeed, TRV-*COMT1* plants had lower ABS/CSm compared with that of TRV plants under normal condition, however, under heat stress no much difference was found between the two genotypes (**Figure 3**). It is likely that at perturbed temperature conditions, absorption may be largely influenced by other factors such as leaf anatomy and arrangement of the chloroplast, and thus light absorption was not consistent with the change in chlorophyll content under heat stress (Mand et al., 2013). Consistent with the light absorption, light trapping indicated by TR/CSm decreased in *COMT1*-silenced plants under both normal and heat stress conditions. ET/CSm indicates the reoxidation of reduced Q_A_ through electron transport over a cross-section of active and inactive reaction centers (Force et al., 2003). In the current study, heat stress decreased ET/Cm both in TRV and TRV-*COMT1* plants, possibly by inactivating the reaction centers (**Figure 3**). The number of active reaction centers in PSII cross-section was shown as open circles in the pipeline leaf model of phenomenological energy fluxes. As shown in **Figure 3**, heat stress decreased the density of the active reaction centers (RC/CSm), indicating that the active reaction centers were converted into inactive reaction centers under heat stress that negatively affected the photosynthetic electron transport efficiency in tomato leaves (Li et al., 2018a). The non-photochemical quenching (D/CSm) was unaffected in TRV plants regardless of temperature conditions. However, D/CSm decreased significantly in *COMT1*-silenced plants under heat stress (**Figure 3**). D/CSm contributes to the balance between the light energy absorption by the PSII and energy consumption by the metabolic sinks under abiotic stress (Force et al., 2003; Li et al., 2018a). Failure to dissipate excess energy as heat might enhance reactive oxygen species generation and subsequent damage to photosynthetic apparatus in *COMT1*-silenced plants under heat stress (Li H. et al., 2016).

Photosynthetic electron transport is vital for sustaining optimal photosynthetic rate and warranting an effective energy flow for plant growth, development, and stress response (Ye et al., 2013). In the present study, heat stress decreased quantum yield of both PSI and PSII in both TRV and TRV-*COMT1* plants. However, the difference between TRV and TRV-*COMT1* plants was only significant for Y(II), but not for Y(I). In general, PSII is more sensitive to heat stress than PSI (Li H. et al., 2016). For example, a severe heat stress (43°C) has no significant effect on PSI photochemical capacity in sweet sorghum (Yan et al., 2013). Therefore, the significantly decreased Y(II) and ETR(II) values in *COMT1*-silenced plants indicate a more severe damage to PSII by heat stress as compared to that of TRV plants. Notably, heat stress also decreases the maximal photochemical efficiency of primary photochemistry (*F*_v_/*F*_m_) in a range of plant species (Tan et al., 2011). Consistent with the Y(II), *F*_v_/*F*_m_ was decreased significantly by the heat stress in TRV-*COMT1* plants compared with that of TRV plants. The decreased PSII activity causes imbalance between the generation and utilization of electrons, leading to photoinhibition (Takahashi and Murata, 2008; Li H. et al., 2016). However, exogenous melatonin pretreatment improved the *F*_v_/*F*_m_ and *A*_sat_ in *COMT1*-silenced plants and their values were more or less equal to that of TRV plants under heat stress (**Figure 5**). These data clearly indicate that endogenous melatonin deficiency due to *COMT1* silencing made the tomato plants more prone to heat stress; however, supplementation of melatonin by exogenous application alleviated heat-induced photosynthetic inhibition, suggesting a vital role of melatonin in maintaining photosynthetic capacity under stressful conditions.

## Conclusion

In conclusion, the present study showed that suppression of melatonin biosynthesis by silencing of *COMT1* led to significant reduction in photosynthetic pigment contents and CO_2_ assimilation under heat stress. Melatonin deficiency-induced reduction in photosynthesis under heat stress was attributed to both stomatal and non-stomatal factors. The pipeline leaf models of phenomenological energy fluxes clearly indicate that melatonin deficiency affected energy absorption and distribution as evidenced by the decreased light absorption, trapping, electron transfer, heat dissipation, and density of active reaction center per cross-section in *COMT1*-silenced plants. Further analysis of chlorophyll fluorescence parameters revealed that PSII was more affected than PSI in *COMT1*-silenced plants by heat stress. However, exogenous melatonin could recompense the *COMT1* silencing-induced reduction in photosynthetic capacity by improving PSII activity in tomato plants. These findings provide genetic evidence in support of a vital role of melatonin in sustaining photosynthetic activity under perturbed temperature conditions in tomato plants, and thus may have useful implication in horticultural crop management in the face of climate change.

## Author Contributions

GA and WX planned the research and performed the experiments. WX, GA, AL, and SC analyzed and discussed the data. GA and WX wrote the article with contribution from other authors.

## Conflict of Interest Statement

The authors declare that the research was conducted in the absence of any commercial or financial relationships that could be construed as a potential conflict of interest.
