# Supplementary figures and images for: COMT1 Silencing Aggravates Heat Stress-Induced Reduction in Photosynthesis by Decreasing Chlorophyll Content, Photosystem II Activity, and Electron Transport Efficiency in Tomato
Source: Front Plant Sci. 2018 Jul 17;9:998. doi: 10.3389/fpls.2018.00998 (PMC6056654; doi:10.3389/fpls.2018.00998)

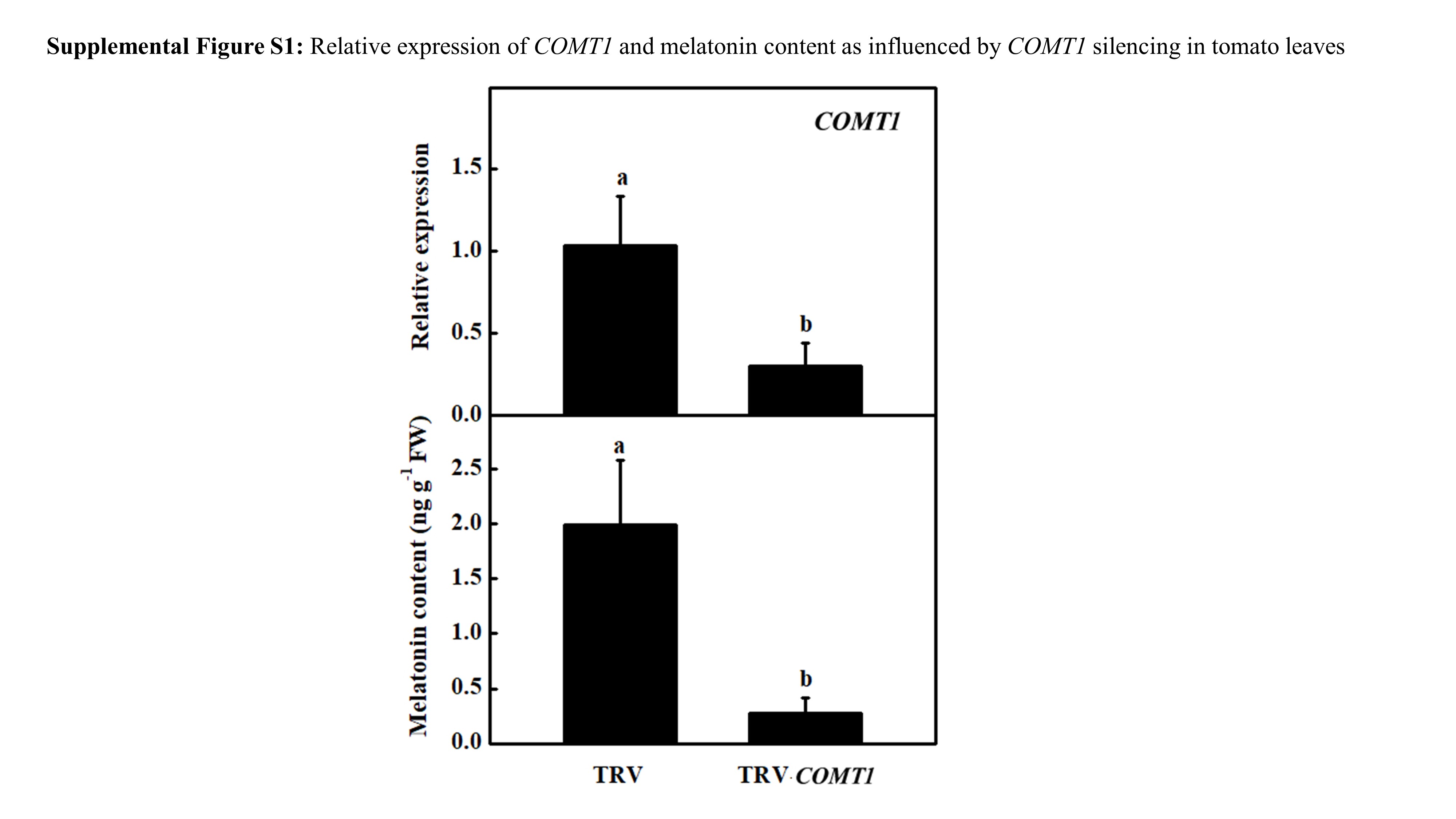

Supplement: Supplementary file 1 [file Image_1.JPEG]
