# Supplementary material for: COMT1 Silencing Aggravates Heat Stress-Induced Reduction in Photosynthesis by Decreasing Chlorophyll Content, Photosystem II Activity, and Electron Transport Efficiency in Tomato
Source: Front Plant Sci. 2018 Jul 17;9:998. doi: 10.3389/fpls.2018.00998 (PMC6056654; doi:10.3389/fpls.2018.00998)
Supplement: Supplementary file 2 [file Table_1.doc]

**Supplementary Table S1.** The formulae and illustrations of JIP-test parameters.

| Formulae and terms | Illustrations |
| --- | --- |
| V_K_=(F_300μs_-F_0_)/( F_m_-F_0_)  V_j_=(F_j_-F_0_)/(F_m_-F_0_)  M_0_=4(F_300μs_-F_0_)/(F_m_-F_0_)  S_m_=(Area)/ (F_m_-F_0_)  φ_P0_=F_V_/F_m_=[1-(F_0_/F_m_)]  φ_E0_=[1-(F_0_/F_m_)]ψ_0_  ψ_0_=(1-V_J_)  ABS/CS_m_≈F_m_  TR/CS_M_=φ_P0_ (ABS/CS_m_)  ET/CS_m_=φ_E0_ (ABS/CS_m_)  RC/CS_m_=φ_P0_ (V_J_/M_0_) (ABS/CS_m_) | Relative variable fluorescence at step K  Relative variable fluorescence intensity at the J-step  Approximated initial slope of the fluorescence transient  Normalized total complementary area above the O-J-I-P transient (reflecting multiple turnover Q_A_ reduction events)  Maximum quantum yield of primary photochemistry (at t=0)  Quantum yield of electron transport (at t=0)  Probability that a trapped exciton moves an electron into the electron transport chain beyond Q_A_^-^ (at t=0)  Absorption flux per CS, approximated by F_M_  Trapped energy flux per CS  Electron transport flux per CS  Density of RCs (QA-reducing PSII reaction centers) |

**Supplemental Table S2.** Primers for generating VIGS vector construction.

| **Gene** | **Encoding protein** | **Accession numbers** | **Forward primer** |
| --- | --- | --- | --- |
| *COMT1* | Caffeic acid *O*- methyltransferase | Sl03g080180 | F: 5’-TGCTCTAGAGCTTTCTTGTTCGCTAT-3’  R:5’-CGCGGATCCGTCCATCAGGGAGTGTC-3’ |

The genes are identified from the Sol Genomics Network (<http://solgenomics.net/>).

**Supplemental Table S3. Genes and primers used for qRT-PCR assays**

| **Gene** | **Encoding protein** | **Accession No.** | **Primer pairs** |
| --- | --- | --- | --- |
| *Actin* | *Actin* | Sl11g005330 | F: 5’-TGTCCCTATTTACGAGGGTTATGC-3’ |
|  |  |  | R: 5’-AGTTAAATCACGACCAGCAAGAT-3’ |
| *COMT1* | Caffeic acid *O*- methyltransferase | Sl03g080180 | F: 5’- CCGATCACCACCACCTTATCC-3’ |
|  |  |  | R: 5’-CCACCAACATCAACAATGGA-3’ |
|  |  |  |  |
